# Children’s visuospatial memory predicts mathematics achievement through early adolescence

**DOI:** 10.1371/journal.pone.0172046

**Published:** 2017-02-13

**Authors:** Yaoran Li, David C. Geary

**Affiliations:** 1 Institute for Entrepreneurship in Education, University of San Diego, San Diego, California, United States of America; 2 Department of Psychological Sciences, University of Missouri, Columbia, Missouri, United States of America; 3 Interdisciplinary Neuroscience Program, University of Missouri, Columbia, Missouri, United States of America; Universiteit Utrecht, NETHERLANDS

## Abstract

A previous study showed that gains in visuospatial memory from first to fifth grade predicted end-of-fifth grade mathematics but not reading achievement, controlling other factors. In this follow up study, these relations were assessed from sixth to ninth grade, inclusive (n = 145). The results showed that growth in visuospatial memory across the elementary school years was related to growth in mathematics achievement after fifth grade, controlling intelligence, the central executive and phonological memory components of working memory, in-class attentive behavior, parental education, and fifth grade mathematics achievement. As found for fifth grade, this relation was not found for reading achievement after fifth grade. In total, the results suggest that visuospatial memory has a unique influence on ease of learning some types of mathematics and that this influence becomes more important across successive grades.

## Introduction

There is a consistent relation between visuospatial ability and mathematics performance and accomplishment in science, technology, engineering and mathematics (STEM) occupations in adults [1–3], but this relation is not consistently found in children [4]. Differences in the mathematical competencies assessed across ages may contribute to this inconsistency. Working STEM professionals can use a combination of mathematical models and visuospatial representations to better understand a variety of physical and biological phenomena, whereas younger children’s mathematical development is largely focused on acquiring competence with number and arithmetic [5]. Skill development in these latter areas is heavily dependent on the central executive component (below) of working memory [6–9], especially the ability to maintain and update information during problem solving [10].

As the focus of mathematical development shifts from number and arithmetic to algebra and geometry across grades, visuospatial ability might become increasingly important for mathematical learning and cognition. For instance, visuospatial ability facilitates the use of diagrams to map out quantitative relations conveyed in algebraic word problems [11], and predicts adolescents’ geometry achievement, controlling intelligence and other factors [12]. In a previous study, we examined the relation between developmental growth in the core working memory systems identified by Baddeley and Hitch [13] and mathematics achievement from first to fifth grade. These systems include the central executive that is critical for the top-down maintenance and manipulation of information stored in two representational systems, the phonological loop and visuospatial sketch pad (hereafter visuospatial memory). We showed that developmental gains in visuospatial memory from first to fifth grade predicted mathematics achievement at the end of fifth grade, controlling prior achievement, intelligence, the central executive and other factors [14].

At the time of this previous analysis, the associated kindergarten to ninth grade longitudinal study was ongoing, and thus we could not assess the relation between visuospatial memory and adolescents’ mathematics achievement. The study has since been completed and we now report on the relation between these students’ visuospatial memory in fifth grade and their mathematics achievement from sixth to ninth grades, inclusive. As with the first study, we control for potential confounding factors that are predictive of developmental gains in mathematical competencies; specifically, the central executive component of working memory, intelligence, speed of processing, and in-class attentive behavior [7, 15–16]. We also conducted parallel analyses on grade-related changes in word reading achievement, to determine if any potential contributions of visuospatial memory were specific to mathematics or predicted achievement gains more generally.

## Materials and methods

### Ethics statement

The study was reviewed and approved by the Institutional Review Board of the University of Missouri. Written consent was obtained from all parents, and all participants provided verbal assent for all assessments.

### Participants

As in our earlier study [14], the participants were children from a prospective study of mathematical development and risk of learning disability who had completed extensive working memory assessments in both first and fifth grades [17]. Two hundred and fifty-four children completed the working memory assessment and the processing speed test in first grade, and 175 of these children completed the same assessments in fifth grade. Thirty of these 175 children were dropped because achievement data were missing for one or more grades in sixth grade to ninth grade. Thus, the final sample included 145 (71 boys) children with no missing data on measures of working memory, intelligence, or achievement. The mean intelligence of this sample was average (M = 102, SD = 15) based on the Wechsler Abbreviated Scale of Intelligence (WASI; [18]). Their kindergarten (M = 104, SD = 12) and sixth grade (M = 97, SD = 17) mathematics achievement were average, and their word reading achievement was above average in kindergarten (M = 114, SD = 14) and average in sixth grade (M = 101, SD = 9). The mean respective ages at the sixth and ninth grade achievement assessments were 12 years, one month (SD = 4 months) and 15 years, 2 months (SD = 4 months). Also, their ages were 7 years, 2 months (SD = 4 months) and 11 years, one month (SD = 4 months) for the first and fifth grade working memory assessments, respectively. Eighty percent of the sample was White, and the remaining children were Black, Asian, or of mixed race. The 30 children we dropped had an average IQ in first grade (M = 97, SD = 10) and average mathematics (M = 98, SD = 15), and word reading (M = 106, SD = 13) achievement in kindergarten.

### Standardized measures

#### Intelligence

The Vocabulary and Matrix Reasoning subtests of the WASI were administered and used to estimate intelligence based on procedures outlined in the manual [18]. For this nationally (U.S.) normed test, the mean score is 100 and the SD is 15.

#### Achievement

Mathematics and reading achievement were assessed using the nationally (U.S.) normed Numerical Operations and Word Reading subtests from the Wechsler Individual Achievement Test-II-Abbreviated [19], respectively. The easier Numerical Operations items assess number discrimination, rote counting, number production, and basic addition and subtraction. More difficult items include multi-digit addition and subtraction, multiplication and division, and rational number problems as well as simple algebra and geometry problems solved with pencil-and-paper. The easier Word Reading items require matching and identifying letters, rhyming, beginning and ending sounds, and phoneme blending. The more difficult items assess accuracy of reading increasingly difficult words. We used the raw scores because they better captured individual differences in achievement growth.

### Working memory

The Working Memory Test Battery for Children (WMTB-C; [20]) is a standardized and normed (U.K.) test of the three core components of working memory. The test consists of nine subtests that assess the central executive (three subtests, α = .75, .68 for first and fifth grades, respectively), phonological memory span (four subtests, α = .78, .77), and visuospatial memory span (two subtests, α = .55, .57). All of the subtests have six items at each span level. Across subtests, the span levels range from one to six to one to nine. Passing four items at one level moves the child to the next. At each span level, the number of items to be remembered is increased by one. We used total number of correct items in the analyses because these are more reliable than span scores.

#### Central executive

The three measures require the child to maintain one set of information in mind, while processing another set of information. Listening Recall requires the child to determine if a sentence is true or false, and then recall the last word in a series of sentences. Counting Recall requires the child to count a set of 4, 5, 6, or 7 dots on a card, and then to recall the number of counted dots at the end of a series of cards. Backward Digit Recall is a standard backward digit span.

#### Phonological memory

The four measures assess the child’s verbatim memory for phonological sounds. Digit Recall, Word List Recall, and Non-word List Recall are span tasks with differing content stimuli; the child’s task is to repeat words spoken by the experimenter in the same order as presented. In the Word List Matching task, a series of words, beginning with two words, is presented to the child. The same words, but possibly in a different order, are then presented again; the task is to determine if the second list is in the same or different order than the first list.

#### Visuospatial memory

Block Recall assesses sequential visuospatial memory as assessed by Kyttala and Lehto [21] and consists of a board with nine raised blocks in what appears to the child as a ‘‘random” arrangement. The blocks have numbers on one side that can only be seen from the experimenter’s perspective. The experimenter taps a block (or series of blocks), and the child’s task is to duplicate the experimenter’s sequence. In the Mazes Memory task, which assesses simultaneous visuospatial memory, the child is presented a maze with more than one solution, and a picture of an identical maze with a path drawn for one solution. The picture is removed and the child’s task is to duplicate the path in the response booklet.

### Processing speed

Speed of encoding and articulating numerals and letters was assessed using the rapid automatized naming (RAN) task [22]. Five letters or numerals are first presented to determine if the child can read the stimuli correctly. The child is then presented with a 5 by 10 matrix of incidences of these letters or numerals and is asked to name them as quickly as possible without making any mistakes [23]. Reaction time (RT in sec) is measured using a stopwatch.

### In-class attentive behavior

The Strength and Weaknesses of ADHD–symptoms and normal-behavior (SWAN) was used as the measure of in-class attentive behavior [24]. The measure includes items that assess attentional deficits and hyperactivity but the scores are normally distributed, based on the behavior of a typical child in the classroom. The nine-item (e.g., ‘‘Gives close attention to detail and avoids careless mistakes”) measure was distributed to the children’s second, third, and fourth grade teachers who were asked to rate the behavior of the child relative to other children of the same age on a 1 (far below) to 7 (far above) scale. Scores were averaged across items and were highly correlated across grades, rs = .70 to.74 (*ps* < .0001), and thus we used the rating that had the most responses (second grade, n = 123).

### Procedure

Achievement tests were administered every spring beginning in kindergarten, the WASI [18] in the spring of first grade, and the RAN every fall beginning in first grade. The majority of children were tested at their school site, and occasionally on the university campus or in a mobile testing van. Testing in the van occurred for children who had moved out of the school district and for the administration of the WMTB-C (e.g., after school). The assessments required between 40 and 60 min.

### Statistical analysis

Sixth to ninth grade raw scores from the achievement tests were analyzed using multilevel modeling. Preliminary analyses showed that the individual-level random intercept and slope effects were significant for all but one variable (ps < .05); the only exception was for the random slope effect when predicting Word Reading scores (p = .26), when all other variables (including school-level effects) were included in the model. In contrast, the school-level (based on sixth grade) random effects were non-significant (ps = .14-.38). To keep the models parsimonious, the main analyses did not include school-level effects. Students’ scores at each time point were nested within each individual, thus a random intercept and a random slope for grades were added to control for random effects, with grade coded sequentially from -3 to 0 for sixth grade to ninth grade, respectively. Intercept values thus represent achievement in ninth grade, and a positive predictor on slope effect indicates that the variable is associated with increases in achievement across grades. Early predictors of ninth grade achievement (intercept) and grade-related changes in achievement (slope) were intelligence, in-class attentive behavior, first-grade working memory, and first-grade RAN RTs. As in our previous study [14], numeral RTs were used to predict Numerical Operations scores and letter RTs were used to predict Word Reading scores. The relation between across-grade gains in working memory and RAN RTs and ninth grade achievement (intercept) and grade-related changes (slope) were estimated with the inclusion of the corresponding fifth grade working memory measures. Also, as a control for sex differences, a boy (coded 1) versus girl (coded 0) contrast was included in the multilevel models. Except for the outcome achievement measures and the sex contrast, all variables were standardized with M = 0 and SD = 1. Finally, we conducted tests that showed the results should not be biased by multicollinearity of the predictors (VIF = 1.0~2.8).

## Results

Descriptive statistics and correlations among the predictor variables and achievement scores are shown in Table 1. The girl vs. boy on intercept contrast effects in the top section of Table 2 show the sex difference in ninth grade achievement, whereas the girl vs. boy on slope effect tests for sex differences in earlier grades relative to ninth grade. The predictor on intercept effects in the middle section of Table 2 indicate the relation between intelligence, in-class attentive behavior, first grade working memory, as well as processing speed and the end of ninth grade achievement. The predictor on intercept effects in the bottom section of Table 2 show the relation between fifth grade working memory and processing speed and the end of ninth grade achievement. The predictor-on-slope effects show the relation between these variables and across grade changes in achievement. To investigate the effects of first-to-fifth grade gains in the three components of working memory as well as processing speed on achievement, we controlled for first grade scores on these measures and focused on the results for the fifth grade measures. These models, with control of sex, intelligence, and in-class attentive behavior are shown as the NO1 and WR1 models in Table 2. To predict the unique contribution of gains in working memory above and beyond fifth grade achievement, we added fifth grade achievement as another control variable (NO2 and WR2 in Table 2). The key results were the same for models that included quadratic slope effects (S1 and S2 Tables).

**Table 1:** Descriptive Statistics and Correlations.

	M	SD	1	2	3	4	5	6	7	8	9	10	11	12	13	14	15	16	17	18	19	20	21
1:1st grade IQ	102.39	14.83	1.00																				
2.In-class attentive behavior	4.89	1.21	0.20	1.00																			
3:1st Grade CE	34.11	9.14	0.37	0.44	1.00																		
4:1st Grade PM	76.07	13.48	0.48	0.37	0.57	1.00																	
5:1st Grade VSM	28.71	6.72	0.27	0.43	0.54	0.43	1.00																
6:1st Grade Number RT	33.76	7.10	-0.13	-0.41	-0.42	-0.23	-0.39	1.00															
7:1st Grade Letter RT	31.53	7.00	-0.18	-0.43	-0.41	-0.32	-0.32	0.76	1.00														
8:5th Grade CE	50.59	9.94	0.47	0.51	0.59	0.50	0.42	-0.39	-0.43	1.00													
9:5th Grade PM	90.74	13.85	0.31	0.32	0.50	0.74	0.32	-0.31	-0.35	0.51	1.00												
10:5th Grade VSM	45.88	8.99	0.25	0.42	0.47	0.30	0.57	-0.32	-0.26	0.49	0.31	1.00											
11:5st Grade Number RT	23.67	5.12	-0.03	-0.37	-0.35	-0.11	-0.23	0.64	0.45	-0.29	-0.18	-0.32	1.00										
12:5st Grade Letter RT	23.29	5.36	-0.14	-0.34	-0.34	-0.22	-0.19	0.54	0.44	-0.34	-0.26	-0.36	0.78	1.00									
13:5th Grade NO	26.58	5.38	0.40	0.45	0.49	0.36	0.37	-0.28	-0.27	0.48	0.28	0.36	-0.20	-0.21	1.00								
14:6th Grade NO	28.66	6.82	0.45	0.51	0.51	0.41	0.43	-0.33	-0.33	0.55	0.33	0.42	-0.23	-0.23	0.82	1.00							
15:7th Grade NO	31.63	8.98	0.45	0.52	0.55	0.36	0.42	-0.36	-0.32	0.54	0.31	0.50	-0.27	-0.28	0.78	0.80	1.00						
16:8th Grade NO	34.19	9.34	0.44	0.46	0.44	0.34	0.40	-0.34	-0.30	0.48	0.28	0.44	-0.26	-0.30	0.72	0.80	0.84	1.00					
17:9th Grade NO	36.08	8.81	0.47	0.49	0.50	0.41	0.43	-0.34	-0.32	0.55	0.33	0.51	-0.25	-0.34	0.69	0.77	0.84	0.87	1.00				
18:5th Grade WR	106.97	8.61	0.53	0.30	0.50	0.49	0.30	-0.42	-0.44	0.56	0.47	0.38	-0.30	-0.44	0.52	0.51	0.53	0.51	0.56	1.00			
19:6th Grade WR	110.18	8.61	0.54	0.27	0.51	0.54	0.26	-0.39	-0.44	0.53	0.52	0.36	-0.24	-0.36	0.45	0.46	0.50	0.48	0.53	0.90	1.00		
20:7th Grade WR	114.16	8.02	0.52	0.31	0.51	0.48	0.27	-0.39	-0.40	0.46	0.46	0.39	-0.28	-0.38	0.50	0.47	0.55	0.49	0.53	0.90	0.91	1.00	
21:8th Grade WR	115.76	7.71	0.55	0.28	0.47	0.48	0.26	-0.38	-0.36	0.47	0.44	0.36	-0.28	-0.38	0.46	0.46	0.53	0.50	0.53	0.87	0.88	0.93	1.00
22:9th Grade WR	118.31	7.38	0.55	0.28	0.53	0.53	0.34	-0.37	-0.40	0.52	0.49	0.39	-0.24	-0.37	0.46	0.46	0.51	0.48	0.54	0.88	0.89	0.91	0.92

Note: *r* > 0.15 *p* < 0.05; *r* > 0.25, *p* < 0.01; *r* > 0.29, *p* < 0.001; CE = Central Executive; VSM = Visuospatial Memory; PM = Phonological Memory; RT = reaction time; NO = Numerical Operations; WR = Word Reading.

**Table 2:** Results for Mixed Models Predicting Growth in 6^th^-9^th^ Academic Achievement.

	Model NO1			Model NO2			Model WR1		Model WR2			
	Estimate	SE	p	Estimate	SE	p	Estimate	SE	p	Estimate	SE	p
**Intercept**	37.81	0.79	< .0001	36.93	0.64	< .0001	119.18	0.74	< .0001	118.06	0.42	< .0001
**Grade**	2.60	0.22	< .0001	2.65	0.22	< .0001	2.54	0.16	< .0001	2.62	0.15	< .0001
**Sex**	-1.68	1.16	0.1499	-0.60	0.94	0.5205	-0.52	1.09	0.631	0.65	0.61	0.2883
**Sex × Grade**	-0.10	0.32	0.7546	-0.17	0.32	0.6064	0.08	0.24	0.7487	-0.01	0.21	0.9652
First Grade Predictors of Achievement												
**Intelligence**	3.10	0.70	< .0001	2.32	0.57	< .0001	2.99	0.64	< .0001	0.90	0.39	0.0197
**In-class attentive behavior**	2.35	0.70	0.001	1.02	0.59	0.0854	0.00	0.66	0.9973	0.21	0.37	0.5698
**1st grade CE**	0.46	0.79	0.5571	-0.57	0.64	0.3799	1.06	0.73	0.1477	0.56	0.41	0.1734
**1st grade PM**	0.19	0.95	0.8417	0.22	0.76	0.7765	-0.28	0.88	0.7472	-0.26	0.49	0.5952
**1st grade VSM**	0.00	0.80	0.9961	-0.02	0.64	0.9737	-0.36	0.73	0.6238	0.07	0.41	0.8566
**1st grade number RT**	-0.45	0.73	0.5401	-0.33	0.59	0.5745						
**1st grade letter RT**							-1.04	0.58	0.0756	-0.04	0.33	0.9094
**Intelligence × Grade**	0.26	0.19	0.1789	0.31	0.20	0.1163	-0.13	0.14	0.3528	0.02	0.13	0.8754
**In-class attentive behavior × Grade**	-0.02	0.19	0.9224	0.06	0.20	0.755	0.06	0.15	0.6751	0.05	0.13	0.7206
**1st grade CE × Grade**	-0.28	0.22	0.1939	-0.22	0.22	0.3242	0.07	0.16	0.662	0.11	0.14	0.4509
**1st grade PM × Grade**	0.30	0.26	0.2469	0.30	0.26	0.2482	-0.18	0.20	0.3487	-0.18	0.17	0.2821
**1st grade VSM × Grade**	0.01	0.22	0.9816	0.01	0.22	0.9777	0.32	0.16	0.0485	0.29	0.14	0.0437
**1st grade number RT × Grade**	0.08	0.20	0.7022	0.07	0.20	0.7292						
**1st grade letter RT × Grade**							0.25	0.13	0.0555	0.17	0.12	0.133
Fifith Grade Predictors of Achievement												
**5th grade CE**	1.00	0.80	0.2114	-0.06	0.65	0.9303	0.03	0.74	0.9707	-1.13	0.42	0.008
**5**^**th**^ **grade PM**	-0.75	0.85	0.3799	-0.01	0.69	0.9863	1.18	0.79	0.1388	0.66	0.44	0.1378
**5th grade VSM**	1.76	0.72	0.0156	2.16	0.58	0.0002	0.40	0.69	0.5652	0.37	0.39	0.345
**5th grade number RT**	-0.78	0.75	0.2995	-1.17	0.60	0.0525						
**5th grade letter RT**							-1.06	0.65	0.1021	0.27	0.37	0.4682
**5**^**th**^ **grade NO**				4.23	0.57	< .0001						
**5**^**th**^ **grade WR**										6.23	0.41	< .0001
**5th grade CE × Grade**	-0.03	0.22	0.8754	0.03	0.22	0.8905	-0.12	0.16	0.4794	-0.03	0.15	0.8317
**5th grade PM × Grade**	-0.07	0.24	0.7637	-0.12	0.24	0.6234	-0.09	0.17	0.5958	-0.06	0.15	0.7211
**5th grade VSM × Grade**	0.46	0.20	0.0226	0.43	0.20	0.0312	-0.13	0.15	0.3892	-0.13	0.13	0.3361
**5th grade number RT × Grade**	-0.37	0.21	0.0795	-0.34	0.21	0.102						
**5th grade letter RT × Grade**							0.03	0.14	0.8312	-0.07	0.13	0.6029
**5**^**th**^ **grade NO × Grade**				-0.26	0.20	0.181						
**5**^**th**^ **grade WR × Grade**										-0.46	0.14	0.0017
**AIC**	2816.5			2751.5			2462.0			2351.7		

CE = Central Executive; VSM = Visuospatial Memory; PM = Phonological Memory; RT = reaction time; NO = Numerical Operations; WR = Word Reading; RT = reaction time.

### Numerical operations

The top portion of Model NO1 indicates that the sex differences were not significant in ninth grade or in earlier grades. The results also indicate that children who scored higher on the intelligence test in first grade (p < .0001) and in-class attentive behavior in second grade (p = .0010) had higher mathematics achievement scores at the end of ninth grade. None of the early measures on slope effects were significant. Critically, the bottom portion of Model NO1 indicates that the visuospatial memory score in fifth grade was a significant predictor of ninth grade Numerical Operations scores (p = .0156), and the positive slope effect indicates that visuospatial memory was associated with growth in mathematics achievement from sixth to ninth grades (p = .0226), controlling for first grade visuospatial memory, as well as all other predictors in the model.

For Model NO1, fifth graders who were 1 SD above average in visuospatial memory solved, on average and controlling all other variables in the model, 30.39 Numerical Operations problems correctly in sixth grade [37.81+(-3)*2.60 + 1.76+(-3)*.46], as compared to 29.63 problems correctly for students who were 1 SD below average in visuospatial memory [37.81+(-3)*2.60+(-1)*1.76+(-1)*(-3)*.46]. The gap of .76 problems is small in size (d = .11). However, fifth graders who were 1 SD above average in visuospatial memory solved, on average and controlling all other variables in the model, 39.57 Numerical Operations problems correctly in ninth grade, as compared to 36.05 problems correctly for students who were 1 SD below average in visuospatial memory. The gap of 3.52 problems is moderate in size (d = .40).

Model NO2 added the fifth grade Numerical Operations achievement score to Model 1A, as noted. The top portion shows that intelligence remained significant (p < .0001), but the in-class attentive behavior measure did not (p = .0854). The relation between fifth grade visuospatial memory and ninth grade Numerical Operations scores remained significant (p = .0002), as did the slope effect (p = .0312). Notably, though fifth grade Numerical Operations scores predicted the ninth grade Numerical Operations scores (p < .0001), the slope effect was not significant (p = .1810). The latter indicates that fifth grade Numerical Operations scores do not contribute to growth in mathematics achievement across sixth to ninth grades, when all other variables are controlled. This model with working memory variables explained 3.2% more variance than did the same model without working memory variables, following the method introduced by Selya, Rose, Dierker, Hedeker, and Mermelstein [25]. The model with visuospatial memory variables alone (i.e., excluding the central executive and phonological memory variables) explained 2.0% more variance than did the model without them.

### Word reading

The top portion of Model WR1 indicated there was no sex difference in ninth grade word reading achievement, or in earlier grades. Notably, except for intelligence (p < .0001), none of the predictors of Numerical Operations scores predicted Word Reading scores at the end of ninth grade. There was a significant first grade visuospatial memory on slope effect (p = .0485). In addition, there was a marginally significant letter processing speed in first grade on slope effects (p = .0555). These two slope effects both are positive, indicating that students with strong visuospatial memory and fast letter processing speeds showed larger across-grade gains in their reading than did other students. There were no other significant effects.

Model WR2 added fifth grade Word Reading scores to Model WR1. The intelligence on intercept effect remained significant, but was reduced in magnitude (p = .0197). The first grade letter processing speed on intercept effect was no longer significant. Fifth grade Word Reading achievement had positive intercept (p < .0001) but negative slope effects (p = .0017). The latter indicates that students with higher word reading in fifth grade gained less in reading from sixth to ninth grades than did students with weaker reading skills. These results also suggested that fifth grade Word Reading scores mediated (Model WR2) the relations between intelligence and the letter processing speed and Word Reading after fifth grade. The visuospatial on slope effect remained significant (p = .0437). However, when predicting word reading achievement, the model with working memory variables did not explain additional variance (0.0%) above and beyond the model without them.

## Discussion

In our previous study [14], we found that children who had the largest gains in visuospatial memory from first to fifth grade had higher end-of-fifth-grade mathematics achievement than did children with smaller visuospatial gains. The current study extends these results through the end of ninth grade and in fact suggests that the importance of visuospatial memory for mathematics achievement increases from sixth to ninth grade. There are several key findings that highlight the specificity and growing importance of visuospatial memory for mathematics achievement.

The first is that visuospatial memory was the only working memory component to uniquely predict end-of-ninth-grade mathematics achievement and gains in achievement from sixth to ninth grade, controlling other factors (e.g., intelligence, in-class attentive behavior). This does not mean that other components of working memory, especially the central executive [11, 26], are not important for mathematical learning, but rather visuospatial memory emerges as a unique contributor to mathematics achievement after the elementary school years, above and beyond the contributions of other aspects of working memory, other domain-general cognitive abilities, and with control of end-of-fifth-grade mathematics achievement. Moreover, our control of first grade working memory competencies indicated that it was growth in visuospatial memory across the elementary school years that was related to growth in mathematics achievement after fifth grade and adolescents’ mathematical competencies. The implications are that a one-time assessment of visuospatial memory in young children may underestimate the importance of these competencies to their later mathematics achievement, and that instruction that focuses on use of visuospatial representations for mathematical problem solving could be particularly useful for students with relatively poor visuospatial memory [27].

The second key finding was that the result for visuospatial memory was specific to mathematics achievement, not to achievement in general. Our findings here are consistent with other recent studies showing similarities in the cognitive abilities that underlie mathematics and reading achievement, such as intelligence, as well as important differences [28, 29]. In keeping with previous findings, faster speed of letter articulation in fifth grade was associated with more fluent word reading at the end of ninth grade [7, 30]. It was surprising however that phonological memory did not emerge as a predictor of fluency of word reading, given previous findings [29]. It is possible that our control of multiple domain-general abilities and two assessments of working memory resulted in overly conservative estimates of the importance of phonological memory. Indeed, examination of the correlations in Table 1 reveals phonological memory is more strongly correlated with reading achievement than mathematics achievement, whereas visuospatial memory is more strongly correlated with mathematics achievement than reading achievement.

Even with conservative estimates, the gap between fluent and less fluent word readers in fifth grade lessened across subsequent grades, potentially due to a ceiling effect for the reading test. Mathematics achievement in fifth grade also predicted subsequent gains in mathematics achievement, with no across-grade change in the gap between higher and lower achieving students (i.e., a non-significant slope effect). In any case, the key point of the contrast of growth in mathematics and reading achievement was to determine if visuospatial memory was specific to gains in mathematics achievement and not achievement more broadly. Our results suggest that this is indeed the case.

Although our study confirmed the importance of first-to-fifth-grade gains in visuospatial working memory in predicting gains in mathematics achievement through early adolescence, we did not examine how and for which types of mathematical content this relation emerges. It is very likely that visuospatial abilities are more important for learning some types of mathematics (e.g., geometry) than others (e.g., whole number arithmetic). Our use of a standard achievement test that included a mix of item types would then underestimate the importance of visuospatial memory for some areas of mathematics, such as geometry [21], and overestimate it for others, such as complex arithmetic [31]. Moreover, we did not have measures of more complex visuospatial abilities that are also related to mathematics achievement [32]. These measures may be better than visuospatial memory or be additive predictors of growth in mathematics achievement.

Of course given the nature of our correlational longitudinal study, the conclusions here are in need of verification with experimental interventions that target visuospatial memory and visuospatial abilities more broadly [27]. It is also unclear how attrition affected the results, even though the final sample was average in terms of intelligence and mathematics and reading achievement. It is unclear if the relation between gains in visuospatial memory and mathematics achievement is moderated by children’s mathematics competencies or other factors. Despite these caveats, the longitudinal nature of the current study and the control of common predictors of mathematics achievement, such as intelligence and the central executive component of working memory, provide strong evidence that visuospatial memory and especially memory gains across the elementary school years contribute to individual differences in mathematics achievement beyond fifth grade.

## Supporting information

S1 Table Results for Mixed Models Predicting Growth in 6th-9th Math Achievement with Significant Quadratic Slope Effects. (DOCX)

S2 Table Results for mixed models predicting growth in 6th-9th reading achievement with significant quadratic slope effects. (DOCX)
