# Supplementary material for: Children’s visuospatial memory predicts mathematics achievement through early adolescence
Source: PLoS One. 2017 Feb 13;12(2):e0172046. doi: 10.1371/journal.pone.0172046 (PMC5305243; doi:10.1371/journal.pone.0172046)
Supplement: S1 Table — (DOCX) [file pone.0172046.s001.docx]

**Table S1.** Results for Mixed Models Predicting Growth in 6^th^-9^th^ Math Achievement with Significant Quadratic Slope Effects.

|  | Estimate | SE | p |
| --- | --- | --- | --- |
| Intercept | 36.63 | 0.66 | <.0001 |
| Grade | 1.77 | 0.53 | 0.0012 |
| Quadratic grade | -0.29 | 0.16 | 0.0704 |
| Sex on intercept | -0.60 | 0.93 | 0.5188 |
| Sex on slope | -0.17 | 0.32 | 0.6008 |
| Intelligence on intercept | 2.32 | 0.56 | <.0001 |
| In-class attentive behavior on intercept | 1.02 | 0.59 | 0.0841 |
| 1st grade central executive on intercept | -0.57 | 0.64 | 0.3779 |
| 1st grade phonological memory span on intercept | 0.22 | 0.75 | 0.7756 |
| 1st grade visuospatial memory span on intercept | -0.02 | 0.64 | 0.9736 |
| 1st grade RAN numeral RT on intercept | -0.33 | 0.58 | 0.5729 |
| Intelligence on slope | 0.31 | 0.19 | 0.1107 |
| In-class attentive behavior on slope | 0.06 | 0.20 | 0.7513 |
| 1st grade central executive on slope | -0.22 | 0.22 | 0.3167 |
| 1st grade phonological memory span on slope | 0.30 | 0.26 | 0.2409 |
| 1st grade visuospatial memory span on slope | 0.01 | 0.22 | 0.9773 |
| 1st grade RAN numeral RT on slope | 0.07 | 0.20 | 0.7252 |
| 5th grade central executive on intercept | -0.06 | 0.65 | 0.9300 |
| 5^th^ grade phonological memory span on intercept | -0.01 | 0.68 | 0.9863 |
| 5th grade visuospatial memory span on intercept | 2.16 | 0.58 | 0.0002 |
| 5th grade RAN numeral RT on intercept | -1.17 | 0.60 | 0.0515 |
| 5^th^ grade math achievement on intercept | 3.80 | 0.59 | <.0001 |
| 5th grade central executive on slope | 0.03 | 0.22 | 0.8888 |
| 5th grade phonological memory span on slope | -0.12 | 0.23 | 0.6180 |
| 5th grade visuospatial memory span on slope | 0.43 | 0.20 | 0.0287 |
| 5th grade RAN numeral RT on slope | -0.34 | 0.20 | 0.0968 |
| 5^th^ grade math achievement on slope | -1.55 | 0.54 | 0.0042 |
| 5^th^ grade math achievement on quadratic slope | -0.43 | 0.17 | 0.0107 |
| AIC | 2744.6 | | |
