# Supplementary material for: Children’s visuospatial memory predicts mathematics achievement through early adolescence
Source: PLoS One. 2017 Feb 13;12(2):e0172046. doi: 10.1371/journal.pone.0172046 (PMC5305243; doi:10.1371/journal.pone.0172046)
Supplement: S2 Table — (DOCX) [file pone.0172046.s002.docx]

**S2 Table.** Results for Mixed Models Predicting Growth in 6^th^-9^th^ Reading Achievement with Significant Quadratic Slope Effects.

|  | Estimate | SE | p |
| --- | --- | --- | --- |
| Intercept | 117.61 | 0.43 | <.0001 |
| Grade | 1.29 | 0.33 | 0.0002 |
| Quadratic grade | -0.44 | 0.10 | <.0001 |
| Sex on intercept | 0.65 | 0.60 | 0.2802 |
| Sex on slope | -0.01 | 0.21 | 0.9646 |
| Intelligence on intercept | 0.90 | 0.38 | 0.0178 |
| In-class attentive behavior on intercept | -0.12 | 0.38 | 0.7568 |
| 1st grade central executive on intercept | 0.56 | 0.40 | 0.1663 |
| 1st grade phonological memory span on intercept | -0.26 | 0.49 | 0.5890 |
| 1st grade visuospatial memory span on intercept | 0.07 | 0.40 | 0.8542 |
| 1st grade RAN letter RT on intercept | -0.04 | 0.33 | 0.9078 |
| Intelligence on slope | 0.02 | 0.13 | 0.8733 |
| In-class attentive behavior on slope | -0.94 | 0.37 | 0.0107 |
| 1st grade central executive on slope | 0.11 | 0.14 | 0.4433 |
| 1st grade phonological memory span on slope | -0.18 | 0.17 | 0.2741 |
| 1st grade visuospatial memory span on slope | 0.29 | 0.14 | 0.0403 |
| 1st grade RAN letter RT on slope | 0.17 | 0.11 | 0.1266 |
| 5th grade central executive on intercept | -0.69 | 0.43 | 0.1123 |
| 5^th^ grade phonological memory span on intercept | 0.66 | 0.44 | 0.1313 |
| 5th grade visuospatial memory span on intercept | 0.37 | 0.38 | 0.3369 |
| 5th grade RAN letter RT on intercept | 0.27 | 0.37 | 0.4608 |
| 5^th^ grade reading achievement on intercept | 6.23 | 0.41 | <.0001 |
| 5th grade central executive on slope | 1.30 | 0.38 | 0.0007 |
| 5th grade phonological memory span on slope | -0.06 | 0.15 | 0.7165 |
| 5th grade visuospatial memory span on slope | -0.13 | 0.13 | 0.3280 |
| 5th grade RAN letter RT on slope | -0.07 | 0.13 | 0.5968 |
| 5^th^ grade reading achievement on slope | -0.46 | 0.14 | 0.0014 |
| In-class attentive behavior on quadratic slope | -0.33 | 0.11 | 0.0044 |
| 5th grade central executive on quadratic slope | 0.44 | 0.12 | 0.0002 |
| AIC | 2326.7 | | |
